# Social Media Entertainment Among Children in Tema, Ghana: Impact of Restrictions and Associated Factors

**DOI:** 10.1155/ijpe/2396594

**Published:** 2025-06-25

**Authors:** Sylvester Kyei-Gyamfi, Frank Kyei-Arthur

**Affiliations:** ^1^Department of Children, Ministry of Gender, Children and Social Protection, Accra, Ghana; ^2^Department of Environment and Public Health, University of Environment and Sustainable Development, Somanya, Ghana

**Keywords:** children, entertainment, Ghana, social media, Tema

## Abstract

**Aims:** To examine the prevalence of children's social media use for entertainment, sex differences in usage, perceptions of how restricting their access may affect them, the reasons behind these views, and predictors of social media use.

**Methods:** Four hundred children aged 8–17 living in Tema, Greater Accra Region, Ghana, were sampled for the quantitative data, while 59 children were sampled for the qualitative data. Descriptive statistics, Pearson's chi-square test, and binary logistic regression were used to analyze the quantitative study, while the qualitative data were analyzed thematically.

**Results:** Nearly 9 out of 10 children use social media for entertainment. Male children are more likely to use social media for entertainment than girls. The findings suggest that denying children access to social media for entertainment may result in negative consequences such as boredom, a scarcity of entertainment options, a lack of access to the most recent information on entertainment advancements and trends, and missed opportunities for online learning and content creation.

**Conclusion:** This study demonstrates that most children use social media for entertainment, and limiting children's access to social media may lead to information isolation, entertainment deficit, and digital disengagement. Consequently, stakeholders must devise interventions that enhance children's access and responsible use of social media for entertainment. Future studies should examine children's frequency and intensity of social media use and its potential benefits and associated risks.

## 1. Introduction

Before the advent of the internet, children typically engaged in outdoor play. However, with the increasing prevalence of electronic devices such as mobile phones, tablets, and computers, combined with a wide array of digital entertainment options, many children now choose to entertain themselves indoors via social media [1–5]. This shift is mirrored in Ghana, where the lack of a dedicated leisure and recreation policy for children, along with limited access to amusement facilities, has led many children to lose interest in outdoor activities, opting instead for online entertainment [3, 6].

New social media platforms, such as YouTube, Snapchat, and Instagram, entertain their users, including children [7]. Social media makes downloading games, audio, and video easy for entertainment. Some platforms also allow users to showcase their works and talents, which helps them improve their skills and creativity [8–10]. It is important to recognize that children use social media not only for entertainment but also for educational and other cognitive purposes [9]. Social media platforms provide valuable resources that support research and academic pursuits, helping children expand their understanding of the world [11, 12]. These opportunities have driven children to adopt social media as a means of entertainment [9, 13]. Spending excessive time on social media for entertainment, as opposed to engaging in outdoor play, can have adverse consequences for children. Studies have shown that high levels of social media use can undermine adolescents' self-esteem [14, 15], heighten their vulnerability to online peer bullying [16–19], and contribute to the risk of developing social media addiction [20, 21]. For example, Kaplan et al. [17] found that excessive engagement with social media can negatively influence adolescents' self-perception. Furthermore, extensive use of social media is associated with increased risks of sexual harassment, privacy violations, sleep disruptions, reduced learning outcomes, and the receipt of unsolicited friend requests from unfamiliar online users [22–26].

The advent of digital platforms, such as social media, has changed the way individuals communicate, interact, participate, and socialize by utilizing Internet infrastructure [13]. According to Kyei-Gyamfi [9], social media enhances communication and provides information for class assignments. Social media and cell phones have become integral components of the daily lives of individuals, and increasingly, children are accessing the internet at progressively younger ages [6, 9, 27, 28]. In Ghana, the Children's Act, 1998 (Act 560), defines a child as a person below the age of 18 years. This legal definition guides the scope of this study, which focuses specifically on children within this age group. According to Kyei-Arthur et al. [27], many Ghanaian children use smartphones to access the internet. In Ghana, the mean age at which children first start using the Internet is 12, which has decreased in recent years [6]. Research shows that social media is replacing traditional means of communication and changing interpersonal relations in the country. More Ghanaian children, defined here as individuals under 18 years, use social media for entertainment and recreation than two decades ago [3, 29].

Despite the numerous studies on social media in Ghana, these studies have mainly focused on politics [30–34] and tertiary students [35–40]. However, few studies have focused on social media use among children [9, 41]. Furthermore, only Kyei-Gyamfi's [9] study focuses on social media entertainment for children. Thus, there are limited studies on social media entertainment among children. This gap in the literature on Ghanaian children's social media entertainment use, defined in line with the Children's Act, limits a comprehensive understanding of the behavioral, social, and developmental implications of such use. A dearth of studies on social media entertainment among children limits the understanding of the multifaceted nature of social media entertainment use among children in Ghana, which will impact the design and implementation of interventions to enhance social media entertainment use among children. Since social media has become a crucial part of daily life, increasing scientific understanding of this phenomenon is critical. Hence, this study examined the prevalence and sociodemographic differentials of children's social media usage, the prevalence and sociodemographic differentials of children's social media usage for entertainment, sex differentials of types of social media entertainment, the perception of whether denying children access to social media for entertainment would affect them and the reasons for such perception, and predictors of social media use.

## 2. Methods

### 2.1. Study Setting

The study was conducted in Tema, Ghana, a West African country bordered by Burkina Faso to the north, the Gulf of Guinea to the south, Togo to the east, and Côte d'Ivoire to the west. Ghana occupies a total land area of 238,540 km^2^ (92,101 mi^2^) and possesses a coastline length of 539 km (334.9 mi) [42]. With a population of approximately 293,000, the Tema Metropolis is located on the Gulf of Guinea coast, approximately 30 km east of Accra, Ghana's capital [43]. The study participants reside in 59 different residential areas across the TMA. The study focused on children aged 8–17 years who lived in the Tema Metropolis and had access to the internet, whether through personal or shared digital devices. These children constituted the primary participants.

For respondents to be eligible to be recruited to participate in this study, they must meet the following inclusion criteria: (i) They must acquire consent from their parents or legal guardians; (ii) they must be between the ages of 8 and 17; (iii) they must reside within the Tema Metropolitan area; and (iv) they must possess a smartphone, tablet, or computer that is equipped with Internet availability. Children who did not have access to these electronic devices and lived outside the defined study area were not included in the study.

### 2.2. Study Design and Sampling Procedure

The study was designed using a cross-sectional methodology that collect quantitative and qualitative data utilizing convenient and snowball sampling methods. A convenient sampling method was used for this study because the researchers' main aim was to gain an in-depth understanding of children's social media usage rather than generalizing the findings to a larger population. The snowball sampling method helps participants to recommend other eligible respondents for a study. Children often reside with parents or guardians. Since children are in the care of their parents or guardians, this study used the population of parents or guardians to calculate the sample size. Afterward, children of selected parents were recruited to participate in the study. The population of parents in Tema is unknown, and therefore, the sample for the study was calculated using the formula:

 $$\begin{array}{c} n=\frac{{\left(Z-\text{score}\right)}^{2}*\text{StdDev}\left(1-\text{StdDev}\right)}{{\left(\text{Margin of error}\right)}^{2}}, \end{array}$$

where *n* is the sample size, *Z*-score is 1.96 for a 95% confidence interval, standard deviation (StdDev) is 0.5, and margin of error is 0.05.

$$\begin{array}{cc} \; & n=\frac{{\left(1.96\right)}^{2}*0.5\left(1-0.5\right)}{{\left(0.05\right)}^{2}}.\\ n=384.16 \end{array}$$

An estimated 4% nonresponse rate was assumed for the study: 0.04∗384.16. Total sample for study = 384.16 + 15.37. Total sample for study = 399.53. Total sample for study = 400.

A directory of parents residing in Tema generated from an earlier study [44] was used as the initial sampling frame. KoboCollect, an open-source application, was used to design an online self-administered survey. The online self-administered survey was shared with parents on the directory of parents through various channels, including WhatsApp, Internet platforms, and email. A notification message was issued to inform these parents about the study's goals and to seek their consent for their children's participation. This notification was disseminated via WhatsApp or text message, chosen for their convenience in reaching participants. The message included a web link to the online survey, which initially allowed 2 weeks for completion.

Due to a low response rate, this period was extended by an additional 2 weeks before the link was deactivated. Parents were encouraged to discuss the study with their children and assist them in accessing and completing the survey if needed. Importantly, only one child was selected per household; if a parent had multiple children, the eldest was intentionally chosen to prevent multiple responses from the same household and to ensure the questions were age-appropriate.

Using a snowball method, all parents and guardians who participated in the study were encouraged to share the link to the survey with other relatives, friends, and coworkers who were also parents or guardians willing to participate in the study. Some parents even shared screenshots of the last screen/page of the online survey, which had “your response has been recorded” of other parents with whom they shared the survey's link. The snowball sampling method was used because parents are often socially connected with other parents, usually friends [45], which makes it easier for them to refer to other parents with similar characteristics. In total, 400 eligible children aged 8–17 years completed the online survey at the end of the data collection period.

For the qualitative data, 59 children participated in phone interviews (PIs), with one child selected from each of the 59 residential areas within the study communities to capture a diverse range of perspectives on social media usage. Each PI lasted approximately 25–30 minutes and was conducted primarily in English unless the respondent preferred a local language. Two trained field assistants conducted the IPs. These trained field assistants contacted parents in the directory of parents who were not invited to participate in the quantitative part of the study. The children of these parents were interviewed via phone to participate in the study. Prior to each interview, parents were notified about the impending session to confirm the child's willingness and availability to participate. This call was made to ensure that families were prepared for the interview, fostering a supportive environment for the child and minimizing any potential anxiety.

### 2.3. Measurement of Variables

#### 2.3.1. Dependent Variable

The dependent variable was “ever used social media?”, and it was measured by “whether children had ever used social media.” The responses were ‘yes' and ‘no'.

#### 2.3.2. Independent Variables

The independent variables for this study were the sex, age, and education of respondents. The sex of respondents was measured as “male” and “female.” Respondents age was measured as “8–10 years,” “11–13 years,” and “14–17 years.” However, the ages of respondents were recategorised into “8–12 years” and “13–17 years” to address large standard error in the binary logistic regression. With education, it was measured as “primary,” “junior high school (JHS),” “senior high school (SHS),” and “tertiary.” Similarly, the education of respondents was recategorised as “primary” and “JHS and above” to address large standard errors in the binary logistic regression.

### 2.4. Data Collection

This study's data was collected from a sample of children during a 3-month period, culminating in July 2023. The online survey questionnaire covered a wide range of topics, including sociodemographic characteristics, children's knowledge and use of social media, access to social media platforms, preferred types of social media, perspectives on the benefits of social media, use of social media, and the importance of social media for children. The front page of the online survey included a brief explanation of the study's aims, inclusion and exclusion criteria, and a statement about getting participants' consent. On the front page of the online survey, participants (parents, legal guardians, and children) provided their written informed consent by clicking whether they agreed or disagreed to participate in the study. This is required before a participant can start responding to the online survey. Participants who selected “I agree to participate in this study” could access the survey questions. In contrast, those who selected “I do not agree to participate in this study” were sent to the end page of the survey, which had “your response has been recorded.” Thus, they could not respond to the survey questions.

The study followed all ethical principles, and all subjects provided informed consent. All participants' data were anonymised by removing their identifiers, such as their names, email addresses, and phone numbers. This ensured the principles of confidentiality and anonymity were adhered to. Children's participation in the study was of their own volition and consent.

Parents and legal guardians were notified of the study's goals and asked to agree for their children to participate. Participants were sent a link to the online survey. Only a small percentage of online survey participants had finished the questionnaire after 2 weeks, requiring an extension. After 2 weeks, the link was closed. In total, 400 children aged 8–17 participated in the survey, whereas 59 participated in the PIs. PI participants were selected from 59 residential locations in the research communities. A child was chosen from each residential area to ensure a diverse range of viewpoints and experiences about social media usage.

### 2.5. Data Analysis

Given the study's mixed-methods approach, two distinct approaches were used to analyze the data. The qualitative data collected by the PIs was evaluated topically. The study covered three major topics: (i) children's use of social media, (ii) their preferred social media platform, and (iii) the reasons for their choice of a favorite social media platform. The PIs were all recorded, transcribed, and methodically classified based on the study's significant themes. The recorded conversations were transcribed and reviewed to provide a thorough knowledge of the individual's stories. Statements and sentences relevant to the study's themes were assigned codes. The codes representing comparable experiences were organized into core themes. Furthermore, similar core concepts were grouped into organizing themes.

The quantitative data from the online survey was analyzed using the Statistical Package for the Social Sciences (SPSS) Version 25. The quantitative data was examined using descriptive statistics, specifically frequencies and percentages. Pearson's chi-square test was also used to analyze four associations: (i) the association between social media usage and sex, age, and education of the children; (ii) the association between the use of social media platforms for entertainment and sex, age, and education of the children; (iii) what entertainment children use social media platforms for and sex of children; and (iv) whether denying children access to social media for entertainment would affect them. Also, binary logistic regression was performed to examine the predictors of social media use. Statements regarded essential during the PIs have been noted as quotes in the report, providing more explanation to the study.

## 3. Results

### 3.1. Sociodemographic Characteristics of Children

The characteristics of the sample are summarized in Table 1. Table 1 shows equal proportions of male (50.0%) and female (50.0%) children. Also, a high proportion of children were aged 14–17 (60.3%) and enrolled in JHS (36.8%). See Table 1 for more demographic characteristics of children.

### 3.2. Ever Used Social Media

The study examined the prevalence and sociodemographic differentials of children's social media usage. According to the data presented in Table 2, the majority of children (87.5%) involved in this study had ever used social media. More male children (91.7%, *p* value = 0.014) had ever used social media than female children (83.3%). Also, all children aged 14–17 had ever used social media, while 7 out of 10 children aged 11–13 (70.1%, *p* value = 0.000) had ever used social media. Furthermore, all children enrolled in SHS (100.0%, *p* value 0.000) and tertiary educational institutions (100.0%) had ever used social media.

### 3.3. Ever Used Social Media for Entertainment

The children were required to provide information on whether they had ever used social media platforms for entertainment.  Table 3 shows that 9 out of 10 children (90.2%) had ever used social media for entertainment. Regarding the sociodemographic differentials of the use of social media for entertainment, only the sex of children was statistically significant (*p* value = 0.002) with children's use of social media for entertainment. More male children (94.9%, *p* value = 0.002) used social media for entertainment than female children (85.0%).

### 3.4. Sex Differentials of Types of Social Media Entertainment

The children provided information regarding the specific forms of entertainment they engage with on social media platforms. The data presented in Table 4 illustrates that all the various forms of entertainment social media were used for were statistically significant with the sex of children. On the one hand, more male children use social media to listen to and download music (60.2%), learn dance or create dance (46.6%), and play sports and gaming (35.2%) than female children. On the other hand, more female children used social media to watch movies (71.2%), socialize and message (70.0%), view comedy and humorous content (54.4%), and fashion (27.5%) than male children.

The PIs revealed that some children benefit in various ways from using social media for a range of entertainment. Through social media platforms, children can be entertained by watching funny videos, enjoying sports events, and learning dances and how to play entertaining games online. Some of the experiences given by children include the following:

Social media offers a plethora of interesting, entertaining content. Through social media, I can view entertaining videos such as comedy visuals, football highlights, basketball highlights, and dance performances, which enables me to acquire new dancing skills. (PI 1; boy; 14 years)

I liked the YouTube word games and used the tutorials to learn how to play Scrabble. These days, I'm good at school Scrabble events. Without being able to use social media, that probably would not have been possible. (PI 2; girl; 13 years)

In addition to learning how to dance, I utilise social media to produce dance material that I hope will inspire others to learn. There is a cycle of advantages available just on social media that is not available on other conventional media, like TV. (PI 3; boy; 17 years)

I can join online virtual gaming sites through social media and play games with my friends when they are at their various residences. We do not have to be together in person to play. We simply do it online. (PI 4; boy; 11 years)

### 3.5. Perception of Whether Denying Children Social Media Entertainment Would Affect them

**Figure 1:**
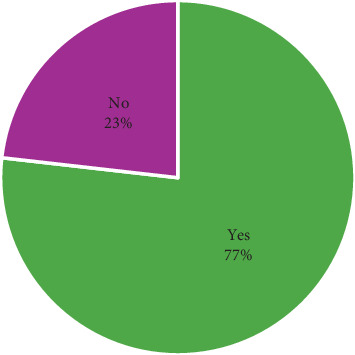
Whether denying children access to social media for entertainment would affect them.

The study additionally explored respondents' perceptions of the potential effects on children when deprived of the opportunity to utilize social media for entertainment. From Figure 1, about 8 out of 10 children (77%) indicated that denying children social media would affect them.

The PIs also sought if children stand to be affected when denied the use of social media to entertain themselves. Some of the views of the children were expressed as follows:

I really do not know how I'll cope if my parents forbid me from using my phone for amusement purposes, such as watching videos on YouTube or TikTok. I find the idea completely inconceivable, and I really doubt it will ever materialize. (PI 5; girl; 15 years)

It is a violation of human rights to deny a child access to social media in this day and age when social media is an essential component of children's lives. No child will be unaffected by this, even if it is not for entertainment. (PI 6; boy; 16 years)

### 3.6. Why Denying Children Social Media Entertainment May Affect Them

Participants were asked to provide an explanation for the potential effect on children when they are deprived of social media entertainment. This was done to determine their perspective on the significance of children's utilization of social media for entertainment purposes.  Table 5 shows that more than half of children (55.8%) reported that depriving children of social media entertainment will deprive them of their favorite entertainment and make life boring. Also, less than 1/10 of children reported that depriving children of social media entertainment will affect their ability to access the most current developments and news in entertainment (7.0%) and adversely affect friends' communications and entertainment sharing (6.2%).

The PIs reiterated similar viewpoints in the survey to validate the quantitative findings. The following statements were provided as evidence to support this claim:

I do not have any other place to get entertained if I do not have access to social media. Social media is presently my only source of entertainment as it provides me with varied opportunities for amusement and recreation. (PI 7; boy; 12 years)

Being denied the use of social media will cost me a lot since there would not be other preferable avenues to easily explore current issues and access relevant information to keep us abreast with the times. (PI 8; girl; 16 years)

Using social media is the best and fastest way to find out what films and songs are currently popular. It has grown so integral to my existence that I cannot fathom a time when I had no access to it. I just do not know what to do if I am denied use of it for entertainment. That, to me, is what social media is all about. (PI 9; boy; 14 years)

Young people gain self-assurance and find ways to beat boredom on social media. You may rest assured that you will be amused and freed of boredom once you reach one of the platforms. To be denied is to have one's enjoyment in life snuffed out. (PI 10; girl; 15 years)

### 3.7. Predictors of Social Media Use

Table 6 presents the results of the binary logistic regression. From Table 6, only the sex of respondents was a significant predictor of social media use (*p* value = 0.003). Precisely, male children were 2.752 times more likely to use social media than female children.

## 4. Discussion

The purpose of this study was to examine the prevalence and sociodemographic differentials of children's social media usage, the prevalence and sociodemographic differentials of children's social media usage for entertainment, sex differentials of types of social media entertainment, the potential effects of denying children access to social media for entertainment, and the underlying reasons why children believe that being denied access to social media for entertainment would affect them. The study also examined the predictors of social media use. Almost 9 out of 10 children said they had used social media platforms at some point, indicating a high usage level among children. The prevalence of social media use in this study (87.5%) is higher than the prevalence of social media use found in studies in the United Arab Emirates (85.7%) [46] and Canada (79.5%) [47]. The difference in the prevalence of social media could be attributed to differences in the characteristics of the study population. For example, Alnjadat et al.'s [46] study examined social media use among medical students aged 16 years and older, while Sampasa-Kanyinga and Lewis's [47] study was among middle and high school children aged 7–12 in Ottawa, Canada. Policymakers and government agencies, such as the Cyber Security Authority, need to educate children on the benefits and risks associated with using social media so that children can harness the benefits of social media while reducing the risks associated with the use of social media.

This study also found that there is a sex differential in the usage of social media among children. Confirming earlier studies, this study found higher social media use among male children than female children [48, 49]. According to Pfeiffer et al. [50], lower social media usage among females could be attributed to cultural norms restricting female behaviors and fear of females disclosing their interest in Internet use to their parents due to negative consequences of Internet use, such as meeting wrong persons. However, some studies found higher social media use among female children compared to male children [51, 52].

The discrepancies in sex differentials of social media use could be attributed to varied factors, including the characteristics of the study population and the year of publication. For instance, Ogundele et al. [51] examined social media use among undergraduate students in Nigeria aged 16 years and older, while Sampasa-Kanyinga and Lewis's [47] study was among middle and high school children aged 7–12 in Ottawa, Canada. The findings of this study highlight the need for sex-targeted interventions to promote Internet use among children in general and social media use in particular. Due to the inconsistent findings on sex differences in social media usage among children, it is essential to perform longitudinal studies. These studies will provide policymakers with a comprehensive grasp of the nuances of children's social media usage, including the evolution of these patterns among the sexes. Such insights are essential for formulating focused and effective policies that address the specific needs of each sex.

The study also found a positive association between children's age, educational level, and social media use. More older children (14–17 years) have ever used social media than younger children (8–13 years). Older children are more likely to get access to the Internet than younger children [27, 53], which may explain their higher usage of social media than younger children. Regarding educational level, as children's educational attainment increased, so did their social media usage. This finding is expected since previous studies have found a positive correlation between children's educational level and internet use [27, 53, 54]. It supports previous studies that found an association between social media usage and educational attainment [55].

Social media is used for entertainment by 9 out of 10 children (90.2%), showing widespread usage. This finding supports previous studies that found that children use social media for entertainment [27, 56]. There is a statistically significant association between using social media for entertainment and the sex of children, with male children (94.9%) reporting higher usage of social media for entertainment compared to female children (85.0%). This finding is expected since more male children use social media compared to female children (Sampasa-Kanyinga et al., 2021; [46]). Though the use of social media for entertainment is good, there is a need for policymakers, including educationalists, to be deliberate about promoting the use of social media for educational purposes, such as for research and schoolwork.

Supporting previous studies [27], children employ social media platforms to partake in an array of entertainment purposes, including but not limited to accessing comedic and enjoyable content, participating in gaming activities, staying informed about sports news, and creating dance-related content; messaging, streaming, and downloading music; acquiring dance skills; and staying abreast of fashion trends. Sex disparities were evident in the findings, with female children exhibiting a preference for watching films, socializing, messaging, consuming comedy, and amusing content. In contrast, male children preferred listening to and downloading music, learning to dance, creating dance content, engaging in sports, and gaming. Prior studies have shown comparable findings, highlighting the distinct usage patterns of social media between males and females [27, 46, 50, 52]. According to Twenge and Martin [52], girls use social media primarily for communication, while boys use it more for pleasure and games. The qualitative findings also demonstrate gender disparities in children's social media usage for entertainment: Females predominantly employ it for fashion-related purposes, while males prefer utilizing it for gaming, sports, learning, and dance creation content.

The study also found that nearly 8 out of 10 children (76.8%) would be affected if they were restricted access to social media for entertainment purposes. Previous studies have found that social media has become part of children's daily lives, and they use it for various activities, including communication, downloading music and videos, and playing video games [27, 46, 52]. Therefore, it is logical to see why children might be more impacted when deprived of social media entertainment.

Over half of the participants indicated that the absence of social media entertainment would result in significant boredom and negatively impact their mental well-being. Other perceived consequences of respondents being denied social media entertainment include a lack of access to entertainment information, sites for sharing new songs, and dance videos. An earlier study by Spina et al. [57] indicated that children who use media gadgets excessively cannot live without them. The current study's responses also suggest that banning children from accessing social media for entertainment may have adverse effects. This discovery is crucial for policymakers, project managers, and others concerned with young people's well-being. The qualitative study suggests that young people need social media, but use can be restricted. Controlling children's social media use should not deprive them of entertainment, as this violates Article 17 of the Convention on the Rights of the Child (CRC) [58]. Article 17 of the CRC addresses the role of the mass media with respect to children's right to information. It requires states parties to enable general access to information and material from a variety of sources, including social media networks. Informing parents, policymakers, and school officials about social media regulation may improve online supervision and prevent children from becoming addicted. This position is consistent with previous research [57].

## 5. Limitations and Strength

It is important to acknowledge the study's limitations to identify areas for improvement in future research. The sample size was limited and confined to children within Tema and may require expansion for a larger sample size in similar research in the future. Due to the study being limited to children in Tema, the study's findings cannot be generalized for the entire country. Also, the study used convenient and snowballing sampling methods, limiting the findings' generalisability. The data collection for this study was conducted through an online survey. Consequently, children who had no access to the internet could not participate in this study.

Additionally, the findings of this study were based on the participants' self-reports. It is possible that the findings of the study could be biased since participants could provide socially desirable responses. Furthermore, the study relied on cross-sectional data, which poses difficulties in generating causal inferences regarding the factors linked to social media usage. This study did not capture the frequency of and time spent on social media by children. Future studies should examine the frequency and time spent on social media use among children. Moreover, parents' population was used to calculate the sample size of the study although the study involved children. Though the sample size calculation was based on the parent population, the actual recruitment and study participants were children of these parents, using the calculated sample size. Although the study has limitations, it is nonetheless significant since it offers a fresh contribution to the limited amount of scholarly research on how children use social media for entertainment.

## 6. Conclusion

This study revealed that the majority of children in Tema have ever used social media. Children's social media use varied by sex, age, and educational level. These findings highlight the need to consider the sociodemographic characteristics of children when promoting social media use among children. Though a significant association existed between children's social media use and age, sex, and educational level, future studies should perform multivariate analysis to examine the relationship between these multiple variables. This will help policymakers and researchers to understand the complex interaction and dependence among the variables.

Also, nearly 9 out of 10 children used social media for entertainment, demonstrating its widespread usage. Social media use for entertainment varies by the sex of children. Children utilize social media for various forms of entertainment, such as watching movies, engaging in social interactions, messaging, streaming and downloading music, accessing comedic and fun content, acquiring dance skills, creating dance-related content, staying updated on sports news, engaging in gaming activities, and keeping up with fashion trends. This study focused on the entertainment use of social media. However, social media has diverse uses, including for educational purposes. Future studies should examine children's use of social media for educational purposes to help policymakers better understand this usage and design appropriate interventions to promote social media use for educational and noneducational purposes among children.

Many children hold the belief that restricting their access to social media for entertainment purposes will have a negative impact on them, potentially resulting in feelings of boredom, a scarcity of entertainment options (such as watching humorous content, playing games, listening to music, and downloading movies), a lack of access to the latest information regarding entertainment advancements and trends, as well as missed opportunities for online learning and content creation. Given that children rely on social media for entertainment, it is imperative for stakeholders to develop interventions that guarantee convenient access and secure utilization of social media for children's entertainment purposes. In situations where there is a need to restrict children's social media use to reduce the risk associated with social media for entertainment, this finding recommends a broader consultation with children to help mitigate the negative effects of restricting children's social media use. Further research is necessary to investigate the extent to which children utilize social media platforms and the potential advantages and risks associated with them. This information will be used to make informed decisions regarding programming and children's social media use policy.

## Figures and Tables

**Table 1:** Sociodemographic characteristics of children.

	**Frequency**	**Percent**
*Sex*		
Male	200	50.0
Female	200	50.0
*Age*		
8–10	72	18.0
11–13	87	21.8
14–17	241	60.3
*Education*		
Primary	112	28.0
JHS	127	31.8
SHS	147	36.8
Tertiary	14	3.5
*Total*	400	100.0

Abbreviations: JHS: junior high school; SHS: senior high school.

**Table 2:** Children who have ever used social media by sex, age, and education.

**Variables**	**Children who have ever used social media**		**p** ** values**
	**Yes (%)**	**No (%)**	
*Sex*			0.014
Male	176 (91.7)	16 (8.3)	
Female	160 (83.3)	32 (16.7)	
*Age*			0.000
8–10	34 (60.7)	22 (39.3)	
11–13	61 (70.1)	26 (29.9)	
14–17	241 (100.0)	0 (0.0)	
*Education*			0.000
Primary	72 (75.0)	24 (25.0)	
JHS	103 (81.1)	24 (18.9)	
SHS	n (100.0)	0 (0.0)	
Tertiary	14 (100.0)	0 (0.0)	
*Total*	336 (87.5)	48 (12.5)	

**Table 3:** Use of social media for entertainment by age, sex, and education of respondents.

**Variables**	**Use social media for entertainment**		**p** ** values**
	**Yes (%)**	**No (%)**	
*Sex*			0.002
Male	167 (94.9)	9 (5.1)	
Female	136 (85.0)	24 (15.0)	
*Age*			0.212
8–10 years	28 (82.4)	6 (17.6)	
11–13 years	57 (93.4)	4 (6.6)	
14–17 years	218 (90.5)	23 (9.5)	
*Education*			0.122
Primary	64 (88.9)	8 (11.1)	
JHS	88 (85.4)	15 (14.6)	
SHS	137 (93.2)	10 (6.8)	
Tertiary	14 (100.0)	0 (0.0)	
*Total*	303 (90.2)	33 (9.8)	

**Table 4:** What entertainment children use social media platforms for by sex.

**Variables**	**Sex of children**		**Total (%)**	**p** ** values**
	**Male** **n** ** (%)**	**Female** **n** ** (%)**		
Watching movies	91 (51.7)	114 (71.2)	205 (61.0)	0.000
Socializing and messaging	83 (47.2)	112 (70.0)	195 (58.0)	0.000
Listening and downloading music	106 (60.2)	51 (31.9)	157 (46.7)	0.000
Comedy/fun features	69 (39.2)	87 (54.4)	156 (46.4)	0.005
Learning dance or creating dance	82 (46.6)	45 (28.1)	127 (37.8)	0.000
Sports and gaming	62 (35.2)	28 (17.5)	90 (26.8)	0.000
Fashion	23 (13.1)	44 (27.5)	67 (19.9)	0.001

**Table 5:** Reasons why denying children the use of social media for entertainment would affect them.

**Reasons**	**Frequency**	**Percent**
Will lead to a deprivation of source of favorite entertainment and making life boring	144	55.8
May experiences depression, which can contribute to the development of mental health conditions	41	15.9
Will be denied access to a site for learning and sharing new dance movements, as well as listening and watching new songs	39	15.1
Will affect the ability to access the most current developments and news in entertainment	18	7.0
It will have an adverse effect on friends' communications and entertainment sharing	16	6.2

**Table 6:** Predictors of social media use.

	**Adjusted odds ratio**	**Standard error**	**Wald**	**p** ** value**
*Sex*				
Male	2.752	0.342	8.762	0.003
Female (RC)				
*Age*				
8–12 (RC)				
13–17	1.173	1.144	0.019	0.889
*Education*				
Primary (RC)				
JHS and above	0.269	1.144	1.320	0.251
*Constant*	6.484	1.150	2.643	0.104

Abbreviation: RC = reference category.

## Data Availability

The data that support the findings of this study are available on request from the corresponding author. The data are not publicly available due to privacy or ethical restrictions.

## Nomenclature

COVID-19: coronavirus disease of 2019
CRC: Convention on the Rights of the Child
DOC: Department of Children
GSS: Ghana Statistical Service
JHS: junior high school
KII: key informant interview
MoGCSP: Ministry of Gender, Children and Social Protection
PI: phone interviews
SPSS: Statistical Package for the Social Sciences
SHS: Senior high school
SSQ: Semistructured questionnaire
TMA: Tema Metropolitan Assembly
UN: United Nations
UK: United Kingdom
UNICEF: United Nations Children Fund
WHO: World Health Organization
